# A Combined Pharmacometrics Analysis of Biomarker Distribution Under Treatment With Standard- or Low-Dose Rivaroxaban in Real-World Chinese Patients With Nonvalvular Atrial Fibrillation

**DOI:** 10.3389/fphar.2022.814724

**Published:** 2022-03-18

**Authors:** Nan Zhao, Zhiyan Liu, Qiufen Xie, Zhe Wang, Zhongyi Sun, Qian Xiang, Yimin Cui

**Affiliations:** ^1^ Department of Pharmacy, Peking University First Hospital, Beijing, China; ^2^ Department of Pharmacy Administration and Clinical Pharmacy, School of Pharmaceutical Sciences, Peking University, Beijing, China; ^3^ Shanghai Qiangshi Information Technology Co., Ltd, Shanghai, China; ^4^ Institute of Clinical Pharmacology, Peking University, Beijing, China

**Keywords:** anti-xa activity, prothrombin time, bleeding, Chinese, population pharmacokinetics, PK/PD, rivaroxaban

## Abstract

**Background:** The rivaroxaban dose regimen for patients with nonvalvular atrial fibrillation (NVAF) is complex in Asia. Given the high interindividual variability and the risk of bleeding caused by rivaroxaban in Asians, the influencing factors and the relationship between outlier biomarkers and bleeding events need exploration.

**Methods:** The integrated pharmacokinetics (PK)/pharmacodynamics (PD) models were characterized based on rich PK/PD data from 304 healthy volunteers and sparse PD [anti-factor Xa activity (anti-Xa) and prothrombin (PT)] data from 223 patients with NVAF. The correlations between PD biomarkers and clinically relevant bleedings in 1 year were explored. The final integrated PK/PD model was used to evaluate the influence of dosage and individual covariates on PD parameters.

**Results:** A two-compartment, linear model with sequential zero-order and first-order absorption was adopted. The dose-specific relative bioavailability (F_1_), diet status, creatinine clearance, and body mass index (BMI) improved the model fit. The apparent systemic clearance was 7.39 L/h, and the central and peripheral volumes were 10.9 and 50.9 L, respectively. The linear direct-effects model with shape factor plus the additive (and/or proportional) error model described the correlation between anti-Xa/PT and plasma concentration. Bodyweight, total cholesterol (TCHO), and diet status were selected as the covariates of the anti-Xa/PT model. Anti-Xa was more sensitive to the increase in rivaroxaban exposure compared with PT. An elevated bleeding tendency was seen with higher peak anti-Xa and PT. For a typical Chinese patient, the peak anti-Xa value (median (5%–95% PI)) of 20 and 15 mg were 309 ng/ml (139–597 ng/ml) and 296 ng/ml (138–604 ng/ml), both median values were within the expected range. For patients with CrCL 30–49 ml/min, the median peak anti-Xa with recommended 10 mg other than 15 mg were within the expected range.

**Conclusion:** Fixed doses of rivaroxaban could be prescribed for patients with NVAF without adjustment for bodyweight, BMI, and TCHO. Randomized studies should be performed to evaluate the efficacy and safety of low-dose rivaroxaban in Chinese patients with NVAF.

## 1 Introduction

Atrial fibrillation (AF) is the most common cardiac arrhythmia that increases the risk of thromboembolic events, congestive heart failure, and mortality (Chao et al., 2018). Rivaroxaban is a direct oral anticoagulant (DOAC) recommended for stroke reduction in patients with nonvalvular AF (NVAF) (Roffi et al., 2016; Lip et al., 2018). One of the main advantages of rivaroxaban is a fixed daily dose with 20 mg qd for patients with NVAF having a creatinine clearance (CrCL) ≥50 ml/min and a low dose of 15 mg for patients with CrCL of 15–49 ml/min (Mueck et al., 2014). However, the dosage regimen of rivaroxaban is complex in Asia. The standard dose was recommended in China. Intriguingly, Japanese people with a low dose of rivaroxaban had similar exposure as Caucasians with a standard dose (Tanigawa et al., 2013). A low dose of rivaroxaban was approved in Japan after the J-ROCKET-AF trial, which showed that a low dose of rivaroxaban was noninferior to warfarin in terms of both efficacy and safety (Hori et al., 2012). In South Korea, over 50% of patients were administered a low dose instead of the standard dose recommended on the label (Lee et al., 2019).

Besides racial differences, interindividual variability (IIV) exists in the pharmacokinetics (PK) and pharmacodynamics (PD) of rivaroxaban, with an interindividual coefficient of variation of 51% for PK parameters (Gouin-Thibault et al., 2017). Asian patients with NVAF had a higher bleeding tendency with warfarin compared with non-Asians (Chiang et al., 2014; Oh et al., 2016). Such high race and IIV and high risk of bleeding require a robust understanding of exposure distribution and quantification of the relationship between outlier exposure and bleeding events.

Routine laboratory monitoring or therapeutic drug monitoring for rivaroxaban is currently not recommended (Cuker, 2016). However, the measurement of rivaroxaban exposure improves patient management in some situations, such as severe bleeding or emergent surgery (Martin and Moll, 2016). Liquid chromatography–mass spectrometry is the gold standard for assessing the plasma concentration of the drug (Gosselin et al., 2018), but it is not accessible in all laboratories. Thus, commercially available anti-factor Xa activity (anti-Xa) chromogenic assay with appropriate drug calibrators may be used for rivaroxaban. Prothrombin time (PT) is an anticoagulant marker sensitive to rivaroxaban (Gosselin et al., 2018), although the sensitivity of PT for anticoagulants differs between reagents (Francart et al., 2014; Volod et al., 2021). Several studies attempted to describe the relationship between PD biomarkers and safety outcomes in a real-world setting. Many others showed that PD biomarkers had a prognostic value for bleeding events (Sakaguchi et al., 2017; Wada et al., 2018; Miklic et al., 2019; Testa et al., 2019); however, others did not show any relationship (Jakowenko et al., 2020). Among these studies, a few of data derived from Asian patients (Sakaguchi et al., 2017; Wada et al., 2018).

The present study aimed to explore the factors contributing to individual PK and PD differences and bleeding events of rivaroxaban. First, a population pharmacokinetics model (popPK) was generated using data from healthy volunteers. Subsequently, PK/PD correlations were investigated in a model based on a mixed healthy volunteer/patient population. The anti-Xa and PT metrics in patients and their links with bleeding events were also explored. In addition, we simulated the PD metrics in the virtual NVAF patient population to compare our simulated results of subgroups to the expected ranges.

## 2 Materials and Methods

### 2.1 Study Design

Studies on healthy Chinese participants (studies 1–5) and patients (study 6) conducted across multiple centers were used for the popPK and PK/PD analysis of rivaroxaban (Supplementary Table S1). All study protocols, their amendments, and informed consent were reviewed and approved by the independent ethics committee of Peking University First Hospital and all the participating sub-central research hospitals.

Studies 1–5 were conducted in a series of bioequivalence in which the pharmacodynamic parameters, including the levels of anti-Xa and PT, were added during the cycles of the reference drug (Xarelto). The participants were confirmed to be healthy prior to the initiation of the study. None of them had been taking any medicine for at least 4 weeks before initiation of the study.

For PK analysis, the venous blood samples were collected pre-dose and then at 16–19 time points after administration of the study drug. For PD analysis, the sampling times were 0, 3, 8, and 12 h after drug administration.

The observational study 6 was conducted in patients regularly taking rivaroxaban (Xarelto) for preventing thrombosis in NVAF. The primary endpoint was the incidence of clinically relevant bleeding (BARC 2, 3a, 3b, 3c, and 5) defined by the Bleeding Academic Research Consortium (BARC) within 1 year (Mehran et al., 2011). Patients who received combined therapy of CYP3A4 strong inhibitors and P-gp inhibitors or CYP3A4 strong inducers and P-gp inducers within 14 days before rivaroxaban treatment were excluded. People with contraindications of rivaroxaban, such as hypersensitivity, active bleeding, previous history of intracranial hemorrhage, and gastrointestinal hemorrhage, in the last 6 months and any major surgeries within 30 days were excluded.

Patients were required to take the study drug daily. After reaching a steady state, 2.7 ml of blood samples were withdrawn before drug administration to determine the trough value and after 3 h of drug administration for the peak value. The follow-up was carried out for 1 year.

### 2.2 Analytical Assay for Blood Concentration and PD Markers

Rivaroxaban concentration was determined using high-performance liquid chromatography–tandem mass spectrometry (HPLC-MS/MS) or liquid chromatography–tandem mass spectrometry (LC-MS/MS) in each subcenter.

The blood samples for anti-Xa and PT detection were centrifuged at room temperature for 15 min at 2500 *g* within 60 min of sampling. The plasma samples were transferred to cryovials and stored at –70°C until PD analysis in one laboratory within 6 months after sampling. PT and anti-Xa were estimated using a Sysmex CS-2100i fully automated multiparameter hemostasis analyzer (Sysmex, Kobe, Japan). PT was determined using Coagulation Method Assay Kits (Thromborel-S, Siemens Healthcare Diagnostics Products GmbH, Marburg, Germany). The anti-Xa activity was assessed using a validated Chromogenic Method Anti-Xa Kit (BIOPHEN DiXaI, HYPHEN BioMed, Neuville sur Oise, France) and reported in rivaroxaban units (limit of detection for rivaroxaban was 0–494 ng/ml).

### 2.3 popPK and PK/PD Model Development

The popPK and PK/PD models were constructed using a nonlinear mixed-effects modeling tool NONMEM (version 7.4.0, ICON Development Solutions, MD, United States), with a first-order conditional estimation using the η–ε interaction method (FOCE INTER) for all model runs. The model was selected based on the likelihood ratio tests, residual analysis, and parameter rationalities. Covariates were added to the basic model if the likelihood ratio test showed a *p* value of ≤0.01 [a change in the objective function value (OFV) of >6.63 with 1 degree of freedom]. Subsequently, covariates were removed from the full model if the likelihood ratio test showed a *p* value of ≤0.005 (a change in the OFV of >7.88 with 1 degree of freedom). The analysis was conducted using R (version 3.5.1, The R Foundation for Statistical Computing), Xpose (version 4.4.0), and PsN (version 3.2.12) (Keizer et al., 2013).

The popPK model was developed based on data from healthy volunteers. Subsequently, the anti-Xa and PT-time profiles of rivaroxaban for healthy volunteers were modeled with each Bayesian estimated PK parameter (Zhang et al., 2003a; Zhang et al., 2003b). Based on the aforementioned PK/PD model, PD data from patients were included to develop an integrated PK/PD model.

#### 2.3.1 Healthy Participants’ popPK Model

One-compartment and two-compartment models were evaluated to describe the PK of rivaroxaban. The concentrations below the low limit of quantification (LLOQ), mainly distributed at 36 and 48 h, accounted for only 4.15% of the total observations and were excluded from the analysis.

IIV was modeled as an exponential error term. The model parameters were assumed to follow log-normal distributions, and the IIV for each structural parameter was modeled in NONMEM following Eq. 1:

$$\theta_{i}=\exp\left(\theta_{tv}+\eta_{i}\right)$$ (1)

where *θ*_*i*_ represents the parameters for the *i*th individual, *θ*_*tv*_ is the fractional change in the natural log of the typical value of the parameters, and *η*_*i*_ is the random variable with zero mean and variance of *ω* ^2^.

The residual variability was described by an additive plus proportional error following Eq. 2:

$$C_{ij}=C_{pred,ij}*\left(1+\varepsilon_{1,pred,ij}\right)+\varepsilon_{2,pred,ij}$$ (2)

where *C*_*ij*_ is the *j*th observed plasma concentration of individual *i*; *C*_*pred,ij*_ is the *j*th model predicted value (plasma concentration) for individual *i*; and *ε*_*1,pred,ij*_ and *ε*_*2,pred,ij*_ are normally distributed residual random errors with a mean of 0 and variances of *σ*_*1*_^*2*^ and *σ*_*2*_^*2*^, respectively.

The covariate analysis was conducted after the selection of the base model. Since varied doses affected the absorption of rivaroxaban, the dose effects were introduced as covariate *a priori* into the base popPK model.

Subsequently, age, sex, body weight (BW), body mass index (BMI), diet status effect, and CrCL were evaluated as covariates on the apparent volume of the central compartment (Vc/F), apparent systemic clearance (CL/F), and zero-order absorption duration of depot compartment (D_1_).

Continuous covariates or categorical covariates were included in the model according to the following Eq. 3 or Eq. 4, respectively:

$$\theta_{i}=\exp\left(\theta_{tv}+\theta_{COV}*\log\left(\frac{COV_{i}}{COV_{pop}}\right)+\eta_{i}\right)$$ (3)

$$\theta_{i}=\exp\left(\theta_{tv}+IND*\theta_{COV}+\eta_{i}\right)$$ (4)

where *θ*_*i*_ represents parameters for the *i*th individual, *θ*_*tv*_ is the fractional change in the natural log of the typical value of the parameters, *θ*_*COV*_ is the fractional change in the natural log of the typical value of the covariate parameters, and *η*_*i*_ is the random variable with zero mean and variance of ω^2^. *COV*_*i*_ is the continuous covariate, and *COV*_*pop*_ is the median of a continuous covariate. *IND* = 1 if the categorical covariate is present, and *IND* = 0 if the categorical covariate is absent.

#### 2.3.2 Healthy Participants’ PK/PD Modeling

PT and anti-Xa data were modeled separately using individual empirical maximum *a posteriori* probability Bayes PK parameter estimates as inputs. The PK/PD structural models were developed from the direct-effect linear or *E*_max_ model.

The effects of demographic factors, liver function, renal function, blood lipid, and smoking history were systematically evaluated in a stepwise forward selection, followed by a backward elimination process.

#### 2.3.3 Integrated PK/PD Modeling

An integrated PK/PD model was developed using both the healthy and NVAF data. The structural model was not modified during the course of combined analysis because the patient study provided only sparse data. The renal function was evaluated as a covariate on the apparent systemic clearance (CL/F).

### 2.4 Model Evaluation

A bootstrap resampling technique was used for model validation. The PK/PD profiles were simulated 1,000 times and compared with the observed data to evaluate the predictive performance of the model. The final PK and PK/PD models were evaluated using standard goodness-of-fit (GOF) plots. The shrinkage of the variability terms was calculated to provide additional information on the properties of the model fit. The predictive performance was evaluated by simulating the data based on the final model and conducting a prediction-corrected visual predictive check (pc-VPC) between observed and simulated data.

### 2.5 Exploratory Exposure-Response Evaluation for Bleeding Endpoint

The correlation between rivaroxaban PD parameters and frequency of clinically relevant bleedings in patients with AF was explored. The subject-specific predictions of the steady-state peak and the trough for PD markers obtained from the empirical Bayes’ predictions were used in the exposure-response (E-R) analyses. The bleeding endpoint was evaluated as a binary outcome (yes/no). The boxplots of PK/PD stratified by bleeding events were generated. The probability of bleeding events was calculated and plotted against PD after classifying the patients according to quantiles of PD values. If an E-R trend was observed, further analysis with linear logistic regression models was used to assess the significance of exposure after incorporating baseline covariates as potential predictors of the probability of events.

### 2.6 Model-Based Simulation

The final population PK/PD model was used to evaluate the influence of dosage and individual covariates on PD parameters. Each simulation was replicated 1,000 times. The simulated PD markers were evaluated based on the calculation of the median and 90% prediction intervals (PI) of the trough and peak at steady state.

## 3 Results

### 3.1 Participant Characteristics

A total of 304 healthy participants provided 4,726 concentration observations for PK evaluation and 1,213 data points for PT and 861 for anti-Xa. A total of 408 and 392 data points were obtained for PT and anti-Xa from 223 patients, respectively.

Table 1 summarizes the relevant demographic and covariate information. For healthy volunteers, rivaroxaban doses were 10, 15, and 20 mg, and fasting and postprandial conditions were evenly distributed. For patients, doses mainly ranged from 10 mg once daily (qd) to 20 mg qd. Further, 68 out of 185 patients with CrCL ≥50 ml/min were prescribed 15 mg or 10 mg, and 9 out of 35 patients with CrCL <50 ml/min were recommended 10 mg or 5 mg. Most parameters of patients with AF were significantly different from those of healthy volunteers; especially, patients with AF had lower CrCL compared with healthy volunteers (78.4 vs. 111 ml/min).

**TABLE 1:** Baseline characteristics of subjects included in the model.

Variables	Healthy volunteers	NVAF patients
n	304	223
Total number of PK observations	4,726	0
Total number of PT observations	1,216	408
Total number of Anti-Xa observations	861	392
Dose, mg(n) and regimen	10 mg (*n* = 185), 15 mg (*n* = 72), 20 mg (*n* = 47), single dosing	5 mg (*n* = 1), 10 mg (*n* = 28), 15 mg (*n* = 69), 20 mg (*n* = 124), 30 mg (*n* = 1), qd
Diet status, n (%)		
Postprandial	137 (45.1%)	101 (45.29%)
Fasted	167 (54.9%)	106 (47.53%)
Male, n (%)	202 (66.4%)	118 (52.9%)
Age, years	30 (18–62)	70 (34–91)
Body height, cm	166 (147–189)	165 (140–192)
Body weight, kg	62.8 (47.0–83.0)	68.2 (38–112)
Body mass index, kg/m^2^	22.8 (19.0–26.1)	24.9 (16.2–38.8)
Creatinine, umol/L	74.7 (16.3–131)	72.0 (33.0–147)
CrCL, ml/min	103 (71.5–568)	76.3 (26.1–178)
ALT, U/L	14 (3–58)	19 (7–120)
AST, U/L	17.0 (7.50–39.0)	20 (12–265)
ALP, U/L	65.0 (30.0–153)	70 (17–139)
HGB, g/L	150 (109–190)	133 (93.0–180)
TG, mmol/L	1.06 (0.290–4.00)	1.19 (0.390–6.91)
TCHO, mmol/L	4.30 (2.66–6.11)	3.96 (1.94–7.92)
CHA_2_DS_2_-VASc	-	3 (0–7)
HAS-BLED	-	2 (0–4)

ALP, alkaline phosphatase; ALT, alanine transaminase; AST, aspartate transaminase; CrCL, creatinine clearance; HGB, hemoglobin; PK, pharmacokinetics; PT, prothrombin time; qd, once daily; n, number of subjects/patients; TCHO, total cholesterol; TG, triglyceride.

Data are presented as median (range) unless stated otherwise.

### 3.2 Heathy Participants’ popPK and PK/PD Modeling Analysis

#### 3.2.1 Heathy Participants’ popPK Model

A two-compartment, linear model with sequential zero-order and first-order absorption described the PK of rivaroxaban (Supplementary Figure S1), similar to the previous rivaroxaban model (Mueck et al., 2007).

The inclusion of relative bioavailability (F_1_) and a dose-specific effect of F_1_ improved the fit of the model; the F_1_ of 15 mg was 13.3% and that of 20 mg was 39.2% smaller than that of 10 mg (dose-specific factor = 1). The zero-order absorption (D_1_) duration was 0.101 h with a lag time (ALAG_1_) of 0.164 h. The absorption rate coefficient (Ka) was estimated as 0.406 (L/h). F_1_ and D_1_ of postprandial participants were 24.4% and 4.90-fold greater than those of fasted participants, respectively. The Ka was 0.8294-fold in fed participants than in fasted participants, and the CL/F was estimated as 9.1 L/h. The estimates of apparent volume (Vc/F) and apparent volume of distribution of the peripheral compartment (Vp/F) were 10.9 and 50.9 L, respectively. The inclusion of the covariate BMI decreased the IIV in Vc/F from 57.0% to 53.8%. The proportional and additive residual variability of the model was 21.0% and 1.87 ng/ml, respectively. The *η* shrinkages were reasonable (<30%) with the exception of 37.99% for F_1_. The final population estimates for PK parameters are shown in Table 2. The GOF plots with both population predictions and individual predictions for healthy participants’ PK are shown in Supplementary Figure S2. The pc-VPCs for popPK model of healthy participants are shown in Supplementary Figure S3. Overall, the PK model predictions were in agreement with the observed plasma concentration data.

**TABLE 2:** Final population estimates for parameters of healthy volunteers model and integrated model.

Parameter	Healthy volunteers model		Integrated model	
	Estimates (RSE%)	IIV% (RSE%)	Estimates (RSE%)	IIV% (RSE%)
PopPK				
Ka (1/h)	0.406 (3.12)	—	0.406, FIX	—
D_1_ (h)	0.101 (20.6)	183 (8.91)	0.101, FIX	183, FIX
ALAG_1_ (h)	0.164 (1.74)	-	0.164, FIX	—
CL/F (L/h)	9.1 (2.50)	21.9 (11.0)	7.39 (2.33)	47.1 (3.07)
V_c_/F (L)	10.9 (4.85)	53.8 (6.10)	10.9, FIX	53.8, FIX
Q/F (L/h)	4.40 (5.07)	77.9 (7.08)	4.4, FIX	77.9, FIX
V_p_/F (L)	50.9 (4.78)	68.1 (8.03)	50.9, FIX	68.1, FIX
10 mg specific factor of F_1_	1, FIX	15.5 (17.1)	1, FIX	15.5, FIX
15 mg specific factor of F_1_	0.867 (24.3)	—	0.867, FIX	—
20 mg specific factor of F_1_	0.608 (6.10)	—	0.608, FIX	—
Impact of postprandial status on F_1_	0.244 (12.1)	—	0.244, FIX	—
Impact of postprandial status on D_1_	4.90 (15.7)	—	4.90, FIX	—
Impact of postprandial status on Ka	0.830 (19.7)	—	0.830, FIX	—
Impact of CrCL on CL/F	—	—	1.84 (5.72)	—
Impact of BMI on V_c_/F	1.36 (31.1)	—	1.36, FIX	—
Proportional residual error (%)	21.0 (0.766)	—	21.0 (0.648)	—
Additive residual error (ng/ml)	1.87 (3.89)	—	1.95 (7.20)	—
PT				
E_0_ (s)	11.4 (0.5)	6.90 (4.6)	11.4, FIX	6.90, FIX
K_1_ (s/(ng/ml))	0.00180 (17)	24.9 (7.7)	0.00180, FIX	24.9, FIX
P_1_	1.37 (2.3)	—	1.37, FIX	—
Effect of bodyweight on E_0_	−0.159 (22.6)	—	−0.159, FIX	—
Effect of TCHO on E_0_	−0.0794 (32.6)	—	−0.0794, FIX	—
Additive residual error (s)	0.363 (1.85)	—	0.372 (2.55)	—
Anti-Xa				
K_2_	0.513 (8.1)	11.0 (16)	0.513, FIX	11.0, FIX
P_2_	1.10 (1.60)	0, FIX	1.10, FIX	—
Effect of postprandial status on slope	0.116 (22.8)	—	0.116, FIX	—
Proportional residual error (%)	16.6 (7.60)	—	22.0 (3.48)	—
Additive residual error (ng/ml)	13.6 (4.30)	—	12.0 (8.30)	—

#### 3.2.2 Healthy Participants’ PK/PD Modeling

##### 3.2.2.1 PT

A linear direct-effects model with shape factor and additive error optimally described the correlation between PT and plasma concentration. The PD model estimates for healthy volunteers are shown in Table 2. The baseline for PT in this study was 11.4 s, the slope of the correlation between PT and rivaroxaban concentration was 0.0018 s/(ng/ml), and the shape factor was 1.37. The IIV was low: 6.9% in the baseline and 24.9% in the slope. BW and total cholesterol (TCHO) were selected as significant covariates on *E*_0_ in the analysis. The residual (additive) variability remaining in the model was low at 0.363 s.

##### 3.2.2.2 Anti-Xa

A linear direct-effects model with shape factor plus proportional and additive error model best fit the study data. The slope of the correlation between anti-Xa and rivaroxaban concentration was 0.513, and the shape factor was 1.1. The IIV of the slope was 11.0%. The diet status was selected as a covariate on the slope in the analysis. The model’s proportional and additive residual variability remained low at 16.6% and 13.6 ng/ml, respectively.

### 3.3 Integrated PK/PD Model

In the case of patients lacking PK data, the integrated PK/PD model was established based on the assumption that the PK/PD correlation was robust across the healthy volunteers and patients; the variation in the observed data was due to progressive PK. The CrCL of patients influenced the excretion of rivaroxaban and was significantly lower than that of healthy volunteers, and hence the CL/F of the PK model was re-estimated. The other parameters, such as Ka, D_1_, AlAG_1_, Vc, Q, and Vp, were fixed values from the healthy participants’ model.

The CL/F of the integrated model was estimated to be 7.39 L/h, and CrCL was selected as a covariate on CL/F in the analysis. The additive residual variability of the PT model was 0.372 s, while the proportional and additive residual variability of the anti-Xa model was 22% and 12.0 ng/ml, respectively.

**FIGURE 1:**
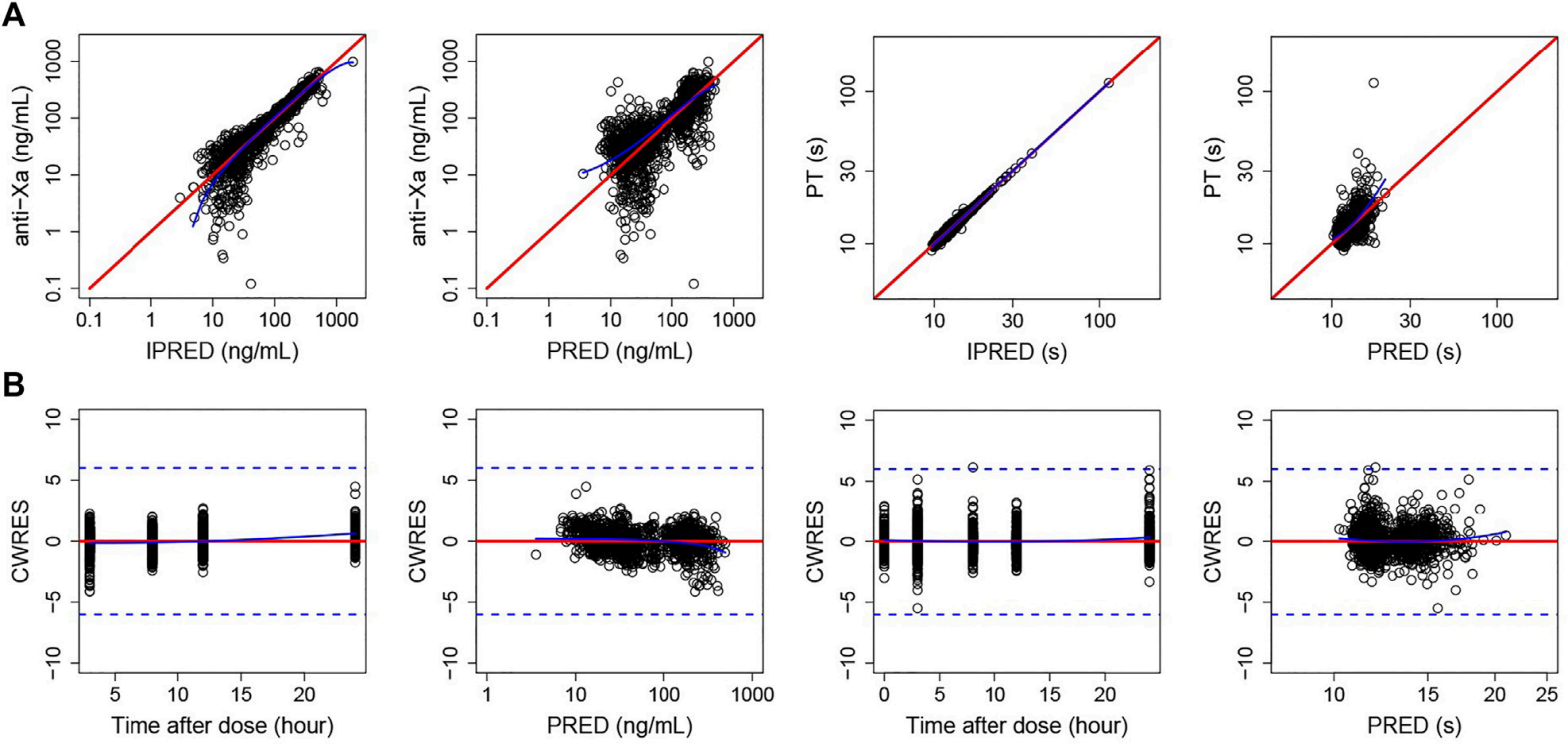
GOF plots for the integrated PK/PD model. **A**: anti-Xa, **B**: PT.

**FIGURE 2:**
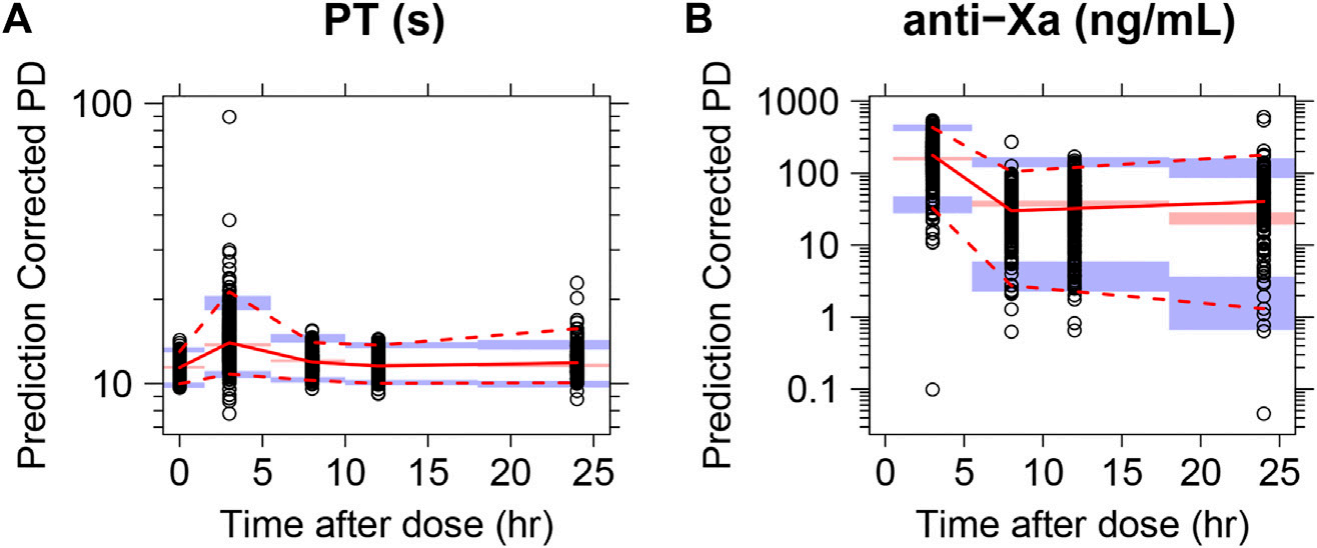
pc-VPC of the integrated PK/PD model. **A**: PT; **B**: anti-Xa.

The GOF plots with both population predictions and individual predictions for integrated PT and anti-Xa models are shown in Figure 1. The population predictions and individual predictions versus dependent variable plots were equally distributed around the 1:1 line. CWRES were randomly distributed around zero across population-predicted values and time. The pc-VPCs of the integrated PD model are shown in Figure 2. The observations were randomly distributed within the calculated 95% PI, demonstrating good overall agreement between the final model and observed data. In addition, nonparametric bootstrap estimations (*n* = 1,000) were performed and confirmed the robustness of the final model and the good precision in estimated parameters (Supplementary Table S2).

### 3.4 Exploratory E-R Analysis

**FIGURE 3:**
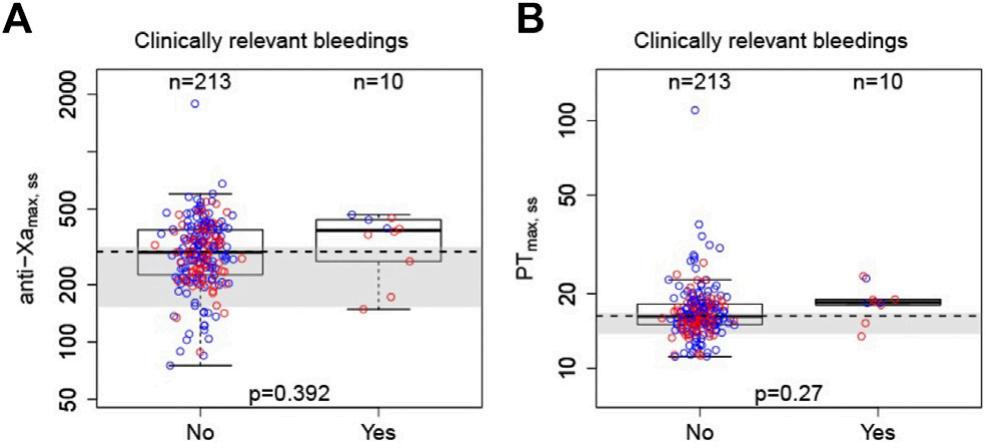
Correlation between peak PT or anti-Xa parameters with bleeding events. **A**: anti-Xa; **B**: PT. Boxes indicate 25th^–^75th percentiles, whiskers represent extension of the box with 1.5 times the interquartile range, and black horizontal lines represent the median. The blue circles are individual predicted values for standard dose or overdose, and the red circles are individual predicted values for low dose. The shaded area represents the expected anti-Xa/PT ranges converted from estimated parameters at a steady state (Steffel et al., 2021).

Clinically relevant bleeding events were seen in 10/223 (4.48%) participants. Figure 3 shows boxplots of anti-Xa and PT of bleedings and non-bleedings. The peaks of anti-Xa and PT in the bleeding group were numerically higher than those in the non-bleedings group. Moreover, the bleeding rates of patients with peak PT and anti-Xa in fourth quartile were higher than those of the others (Supplementary Figure S4). However, the ranges of anti-Xa or PT metrics of bleedings overlapped with the range of the participants without events. No clear correlations were established between PD parameters and safety endpoints.

### 3.5 Exposure Simulation

**FIGURE 4:**
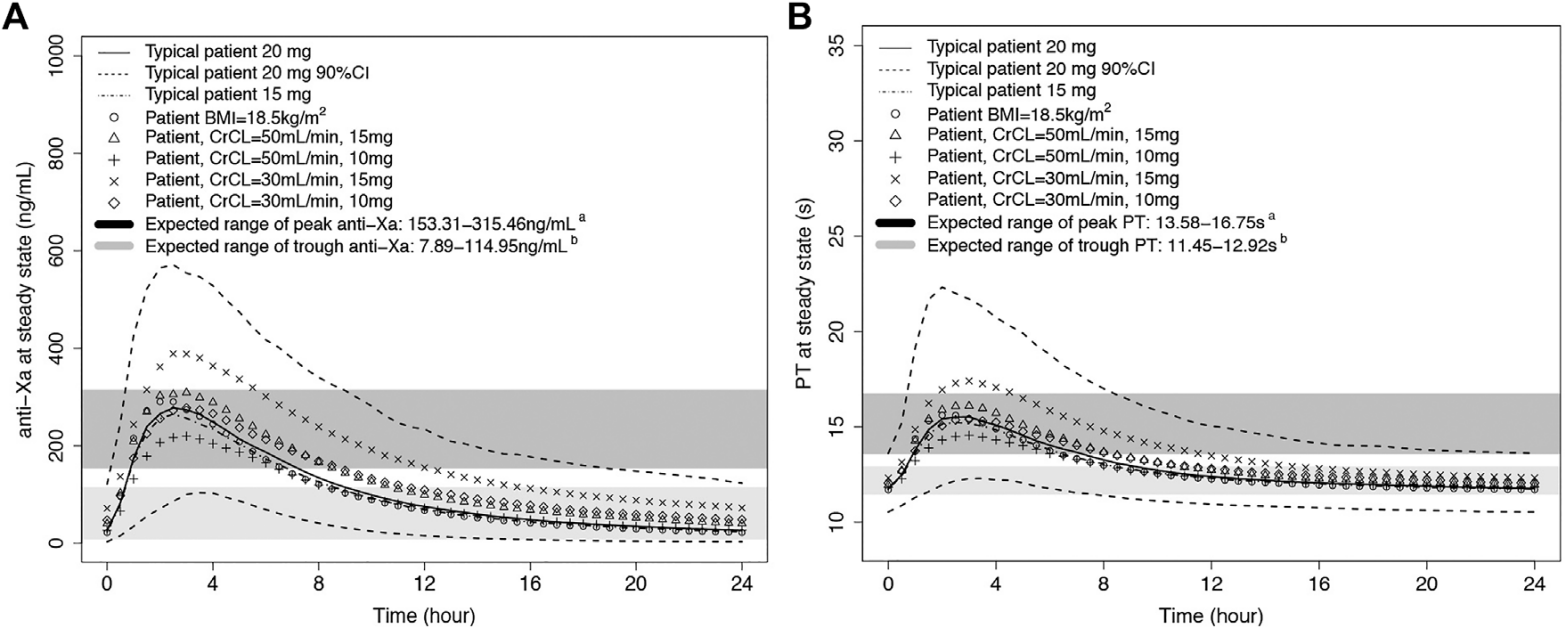
Predicted rivaroxaban PD-time profiles. **A**: anti-Xa-time profile; **B**: PT-time profile. The typical patient has mean characteristics (BMI = 24.7 kg/m^2^, WT = 68 kg, TCHO = 3.81 mmoL/L, CrCL = 75.4 ml/min). **A**: The expected ranges of peak anti-Xa or PT were calculated from the expected plasma peak level of rivaroxaban in NVAF patients (Steffel et al., 2021) by the integrated PK/PD model in our study. **B**: The expected ranges of trough anti-Xa or PT were calculated from the expected plasma trough level of rivaroxaban in NVAF patients (Steffel et al., 2021) by the integrated PK/PD model in our study.

The final integrated PK/PD model was employed, and the PK/PD parameters were predicted at a steady state for each virtual subpopulation (defined by BMI, BW, TCHO, and CrCL). Figure 4 shows anti-Xa and PT simulation results. The final PK/PD models were used to generate real-world typical patient reference value. The peak anti-Xa values [median (5–95% PI)] of 20 and 15 mg were 309 (139–597) ng/ml and 296 (138–604) ng/ml, and the peak PT values [median (5–95% PI)] of 20 and 15 mg were 16.1 s (13.0–23.1 s) and 15.9 s (12.7–22.6 s); both the anti-Xa and PT parameters were within the expected range of standard dose (Steffel et al., 2021). Compared with the typical patient, the population medians of anti-Xa increased by 1.55–12.6% among the predefined low-BMI, low-BW, and low-TCHO groups, indicating minor effects on rivaroxaban exposure. The peak anti-Xa increased by 14.5%–38.5% for patients with CrCL 30–49 ml/min under treatment with rivaroxaban 15 mg, almost out of the aforementioned expected range. The peak anti-Xa distribution would be within the expected range if we reduced the dose of rivaroxaban to 10 mg in patients with CrCL 30–49 ml/min.

## 4 Discussion

In this study, we successfully established an integrated PK/PD model for rivaroxaban based on abundant PK and PD data of Chinese healthy participants and sparse PD data of real-world patients with NVAF. The relative bioavailability of different dosages was explored. The simulation results showed the PD marker distribution under different dosages in Chinese patients.

The lack of PK sampling from patients prevented us from directly modeling the PK/PD in patients. However, based on the established healthy volunteers’ popPK and PD model, we found that the IIVs of PK parameters were much greater than those of PD parameters. In addition, PD values, especially anti-Xa, showed a good linear relationship with increases in plasma concentrations consistent with the International Council for Standardization in Hematology (ICSH) recommendations for the laboratory measurement of DOACs (Gosselin et al., 2018). Thus, it was speculated that the variation in observed data was mainly derived from PK progress. A previous study (Kubitza et al., 2013) indicated that the reduced renal clearance of rivaroxaban was attributed to high area under the curve (AUC) observed in the elderly participants with no clinically relevant effect on *C*_max_. In line with this, the median of CrCL was 29% lower in patients with NVAF than in healthy participants, and the trough anti-Xa and PT values were higher than those of the latter. Therefore, it was anticipated that CrCL, the composite of creatine, weight, age, and sex calculated using Cockcroft–Gault formula (Michels et al., 2010), would affect the PK of rivaroxaban. The data from healthy volunteers and patients were pooled together to inform and stabilize the structure model without any alterations. Moreover, since data in the absorption phase were lacking in patients, the absorption parameters were fixed to population estimates of healthy volunteers, limiting the model predictions in the absorption phase. However, these parameters were expected to influence the derived exposure measures minimally (Kubitza et al., 2013). Also, the meal timing and content do not impact the concentration of rivaroxaban, as assessed by previous clinical pharmacological studies (Kubitza et al., 2006; Stampfuss et al., 2013; Zhang et al., 2017). Subsequently, the integrated model was successfully developed by exploring the PK and PD correlations in healthy individuals and PD data in patients.

We compared the model-estimated population mean value of the PK parameters of rivaroxaban in patients with NVAF across different popPK models (Supplementary Table S3). Most patient models for rivaroxaban were one-compartment models due to the sparsely sampled PK data, which might cause a slight trend toward an underestimation of *C*_max_ (Willmann et al., 2018). The structural PK model in our study established using the abundant PK data from healthy volunteers was a two-compartment model. In agreement with the findings from dedicated PK studies of rivaroxaban (Kubitza et al., 2005a; Zhao et al., 2009) and, as expected, the limited aqueous solubility of Biopharmaceutical Classification System class II substance (Kushwah et al., 2021), the dosage had a highly significant effect on F_1_. We tried a nonlinear absorption model with saturated absorption added to the differential equation to describe the dose effect on F_1_. However, the model was not easily fitted. Then, F = 1/(1 + (DOSE-10)/ID_50_) was used, but we speculated that the estimates of ID_50_ were unreliable for too high ID_50_ values compared with the dosage range. Finally, we referred to previous studies (Mueck et al., 2008; Mueck et al., 2011; Xu et al., 2012) to assign specific factor values to different dose cohorts covering the clinical doses for patients with NVAF, which were best fitted. The relative bioavailability of 15 mg rivaroxaban was 1.43-fold that of 20 mg. The exposure of 15 mg was rarely investigated in previous traditional PK studies (Kubitza et al., 2005a; Kubitza et al., 2005b; Kubitza et al., 2006; Zhao et al., 2009); the investigation of the relative bioavailability of 15 mg was also ignored in some of the models (Mueck et al., 2011; Tanigawa et al., 2013). In the integrated population PK model reported earlier, the relative bioavailability of 15 mg was estimated to be 1.08-fold of 20 mg with a sparse PK sample (Willmann et al., 2018). The gap in F_1_ between 15 and 20 mg in our study was larger than that in the aforementioned study, which might also explain why the AUC of 15 mg in healthy volunteers was similar to that of 20 mg (Supplementary Figure S5), and the distribution of PD metrics in patients using a low dose of 15 mg was similar to that in patients using 20 mg. The diet status also decreased the IIV inherent in the absorption of rivaroxaban, thereby improving the predictability of its PK.

Owning to the medians of the covariates being different across the study populations, we corrected the median covariate values to compare PK parameters among our model and two previous models (Kaneko et al., 2013; Girgis et al., 2014). The CL/F in our study was higher than that of J-ROCKET-AF (mainly Japanese) and lower than that of ROCKET-AF (for patients with CrCL 56.5 ml/min, CL/F was 5.4, 6.1, and 4.6 L/h for Chinese, Caucasian, and Japanese, respectively). The mean value of V (Vc + Vp) estimated in our study was between that of both models. The included covariates were similar across our model and previous models. The renal function was identified as a significant covariate of CL, and size parameters were shown to influence V. A general consistency existed between comparison patients’ data among these ethnicities and healthy participants, which showed that Japanese had an apparent higher dose-normalized rivaroxaban exposure compared with Chinese and Caucasians (FDA, 2022).

A near-linear relationship was found between rivaroxaban concentration and anti-Xa. A linear model plus shape factor reasonably described the concentration–anti-Xa relationship, which was consistent with the previous model (Choi et al., 2016). For PT model, the *E*_max_ model showed too high EC_50_ values compared with the observed plasma concentration range, indicating that PT model was best described by the linear model and was consistent with the previous models (Mueck et al., 2008; Mueck et al., 2011; Kaneko et al., 2013; Tanigawa et al., 2013; Suzuki et al., 2018; Zdovc et al., 2019). TCHO negatively correlated with the *E*_0_ of PT. This phenomenon was consistent with previous findings, wherein high blood lipid levels were related to increased coagulation activity in a normal population (Kim et al., 2015). BW also had the same effect, although the negative correlation between BW and PT was unclear. Notably, our model also showed that the postprandial state had a positive effect on the slope of anti-Xa, indicating that the rate of increase in anti-Xa was greater than that of the increase in plasma concentration in the postprandial state than that in the fasting state, although the causes are yet to be identified. The estimated mean slope (0.0018 s mL/ng for PT and 0.513 for anti-Xa) and P (1.37 for PT and 1.1 for anti-Xa) showed that the sensitivity of anti-Xa toward an increase in rivaroxaban exposure was higher than that of PT. This finding was consistent with the evidence-based guidelines of the ICSH, which favored anti-Xa assays as the test of choice to quantify oral direct FXa inhibitors (Gosselin et al., 2018). The similarity in the relationship of PT and plasma rivaroxaban in J-ROCKET-AF and ROCKET-AF trials and low overall IIV for PT models indicated no significant difference in the relationship between rivaroxaban and plasma concentration among ethnicities (Kaneko et al., 2013; Girgis et al., 2014). The sensitivities of different PT reagents to increases in the plasma concentrations of rivaroxaban were different (Gosselin et al., 2016; Suzuki et al., 2018). Relative to these two models (Supplementary Table S4), our study showed a lower PT reagent sensitivity and consistent with previous study which showed that Thromborel S^®^ in our study was less sensitive than the other four commercially available reagents including Neoplastin Plus^®^ used in these two models (Shimomura et al., 2016).

Assessment of the potential relationship between exposure or pharmacodynamic biomarkers and bleeding events would be meaningful in guiding dose adjustment in specific patients. The post hoc E-R analysis performed in patients with NVAF using data from phase III ROCKET-AF trial showed shallow E-R relationships for both major bleeding and the composite of major or nonmajor clinically relevant bleeding. Patient characteristics such as history of gastrointestinal bleeding appeared to be more important for risk than rivaroxaban exposure (Zhang et al., 2020). However, no clear relationship between the estimated exposure of rivaroxaban and the occurrence of bleeding events was observed in J-ROCKET-AF trial partly due to the limited dose range (Kaneko et al., 2013). Relative to rivaroxaban, a steeper relationship between trough concentration and bleeding risk was reported for edoxaban, another direct factor Xa inhibitor (Salazar et al., 2012).

The relationship between PD biomarkers and clinical outcomes was also evaluated in several studies. Two studies with 156 and 94 patients using multivariable-adjusted Cox models showed that the anti-Xa peak value was independently related to the incidence of hemorrhagic events (Sakaguchi et al., 2017; Wada et al., 2018). A retrospective study with 194 patients showed that the median anti-Xa levels in patients with and without major bleeding were similar. However, the value of anti-Xa in the bleeding group was allowed to be collected in 24 h within the bleeding event, and most of levels were random levels, which might have influenced the conclusion (Jakowenko et al., 2020). A meta-analysis that assessed different PD markers in predicting the clinical outcomes for patients taking DOACs showed that the peak PT (19–25 s) and anti-Xa had a better predictive value on bleeding outcomes for rivaroxaban (Liu et al., 2020). However, the good indicator of thromboembolic events of rivaroxaban was not identified. A small number of bleeding events occurred in our study. We observed an elevated bleeding tendency with high peak anti-Xa and PT. However, the E-R model for the bleeding could not be characterized due to the low incidence and overlapping PD parameters.

Liu et al. provided the expected levels of the mean anti-Xa activity for 10, 15, and 20 mg rivaroxaban using the observed data (Liu et al., 2021). In this study, we applied the advantages of pharmacometrics analysis to find the influencing factors using the aforementioned and some supplementary data to simulate the distribution of PD markers in typical Asian patients. For a typical patient, the peak anti-Xa value (median (5–95% PI)) of 20 mg was 309 (139–597) ng/ml, and the median value was within the expected range for the standard dose (Steffel et al., 2021). The identification of the covariates affecting PK/PD allowed the simulation of several scenarios for patients with various extremes of the covariates. The difference in PD metrics between patients with the extreme value of one characteristic except CrCL was slight, which was in line with previous studies that recommended no dose adjustment except CrCL (Barsam et al., 2017). However, our study showed a wider 5–95% PI owning to the patient data obtained from real-world cases. Accumulating evidence illustrated that patients with NVAF in a real-world setting had a wide range of anti-Xa levels (Samos et al., 2018).

Physicians in Asia tend to prescribe reduced-dose NOACs to patients due to the bleeding concern (Cho et al., 2018); 34.5% of patients in our study were ordered a reduced dosage. The simulation results showed that 15 mg had a PD distribution similar to that of 20 mg, and both peak and trough values were within the expected ranges. For patients with CrCL 30–49 ml/min, the peak anti-Xa values with rivaroxaban 15 mg were almost beyond the expected range. The simulation anti-Xa of 10 mg for patients with CrCL 30–49 ml/min was almost within the expected range, which might benefit patients. Also, the J-ROCET AF trial that used 10 mg o. d. in patients with CrCL 30–49 ml/min demonstrated a strong trend for a decrease in the rate of stroke/systemic embolism with rivaroxaban versus warfarin and a good safety profile. A retrospective study (Chan et al., 2019) included 3,162 patients for comparison of safety and efficacy following ROCKET-AF or J-ROCKET-AF dosage criteria and showed that, for patients with an eGFR of ≥50 ml/(min⋅1.73 m^2^), the risk of clinical events did not differ significantly between the two dosage criteria of rivaroxaban, while for patients with an eGFR <50 ml/(min⋅1.73 m^2^), the ROCKET-AF dosage was associated with a higher risk of major bleeding compared with the J-ROCKET-AF dosage. Thus, we speculated that the similar exposure of 15 and 20 mg and their significantly higher exposure than 10 mg was a potential reason. Our simulation warranted the need for randomized studies to evaluate the efficacy and safety of low-dose rivaroxaban for Chinese patients with NVAF.

Nevertheless, the present study had several limitations. Most importantly, this study was limited by the lack of rivaroxaban concentration in patients; also, the data of concomitant medication and gene polymorphisms might have influenced the PK of rivaroxaban (Mueck et al., 2013; Gouin-Thibault et al., 2017). All of these prevented an adequate evaluation of patient PK. The sparse sampling of PK and PD for patients should be carried in the future for external validation. Second, the pc-VPC of the integrated model suggested overall acceptable predictability of the final model, except for a slight underprediction for low-concentration anti-Xa. These were unlikely to be clinically meaningful because of the inherent loss in precision and accuracy of laboratory assays at low concentrations. Third, the pharmacodynamic effects of rivaroxaban were strongly affected by the type of PT or anti-Xa reagents. Despite several ways to correct the differences in the sensitivity of reagents by adjusting the slope parameter (Yoshioka et al., 2018), the PK/PD models in this study should be carefully interpreted, especially with different reagents. Moreover, the dose-specific effect of F_1_ was only assigned to dosage range for NVAF indication, limiting the simulation of other doses beyond this range and other indications. However, doses over the range of 10–20 mg were rarely used. Finally, The anti-Xa and PT values in the bleeding patients tended to be higher. However, the data should be interpreted cautiously due to the small number of bleeding events. Whether this finding of high anti-Xa and PT translates to a clinically significant difference needs to be validated in a larger study exploring system systemic embolism and bleeding as primary outcomes.

## 5 Conclusion

In this study, we developed a PK/PD model for rivaroxaban based on the data of Chinese healthy participants and real-world patients with NVAF. The final PK/PD models were used to generate a reference biomarker distribution in real-world Chinese patients with NVAF. The peaks of anti-Xa and PT of typical patients treated with 20 mg or 15 mg rivaroxaban were similar and within the expected range. However, for patients with moderate renal impairment, the anti-Xa peak values of rivaroxaban 15 mg were almost beyond the expected range. The simulation results indicated the need for further prospective and randomized studies to evaluate the efficacy and safety of the low-dose rivaroxaban.

## Data Availability

The raw data supporting the conclusion of this article will be made available by the authors, without undue reservation.
