# Supplementary material for: A Combined Pharmacometrics Analysis of Biomarker Distribution Under Treatment With Standard- or Low-Dose Rivaroxaban in Real-World Chinese Patients With Nonvalvular Atrial Fibrillation
Source: Front Pharmacol. 2022 Mar 18;13:814724. doi: 10.3389/fphar.2022.814724 (PMC8971662; doi:10.3389/fphar.2022.814724)
Supplement: Supplementary file 1 [file DataSheet1.docx]

**Supplementary Table S1 Summary of studies included in the rivaroxaban popPK and PK/PD analysis**

| Study | objective | population | N | Rivaroxaban dosing | PK sampling | PD sampling |
| --- | --- | --- | --- | --- | --- | --- |
| 1 | BE | HVs | 56 | 10 mg single dose,  in fasted or fed state | Full PK, 0-48 h | 0, 3, 8, 12 h |
| 2 | BE | HVs | 74 | 10 mg single dose,  in fasted or fed state | Full PK, 0-48 h | 0, 3, 8, 12 h |
| 3 | BE | HVs | 47 | 20 mg single dose,  in fasted or fed state | Full PK, 0-48 h | 0, 3, 8, 12 h |
| 4 | BE | HVs | 55 | 10 mg single dose,  in fasted or fed state | Full PK, 0-48 h | 0, 3, 8, 12 h |
| 5 | BE | HVs | 72 | 15 mg single dose,  in fasted or fed state | Full PK, 0-48 h | 0, 3, 8, 12 h |
| 6 | Real-world study | NVAF patients | 223 | 5, 10, 15, 20 or 30 mg in fasted or fed state | none | Pre-dose, 3 h |

**Supplementary Table S2 the integrated PK/PD model parameter estimates and bootstrap results**

| Parmameters | Final model  Estimate (95% CI) | Bootstrap  （95% CI） |
| --- | --- | --- |
| PK |  |  |
| CL/F (L/hr) | 7.39 (7.10 – 7.77) | 7.39 (7.03 – 7.77) |
| Impact of CrCL on CL/F | 1.84 (1.65 – 2.06) | 1.84 (1.58 – 2.13) |
| IIV of CL/F (%) | 47.1 (44.1 - 49.8) | 46.8 (40.7 - 53.3) |
| Proportional residual error (%) | 21.0 (20.7 - 21.3) | 21.0 (19.3 - 22.6) |
| Additive residual error (ng/mL) | 1.95 (1.81 - 2.09) | 1.96 (1.52 - 2.53) |
| PT |  |  |
| Additive residual error (s) | 0.372 (0.362 - 0.381) | 0.368 (0.326 - 0.414) |
| Anti-Xa |  |  |
| Proportional residual error (%) | 22.0 (20.4 - 23.4) | 21.9 (17.6 - 25.2) |
| Additive residual error (ng/ml) | 12.0 (11.0 – 13.0) | 12.1 (10.7 - 13.7) |

**Supplementary Table S3 Comparison of model-based PK parameters of rivaroxaban across different popPK models**

| Parameter | Our study | ROCKET-AF model(1) | J-ROCKET-AF model(2) |
| --- | --- | --- | --- |
| Ka(h^-1^) | 0.406 | 1.16 | 0.617 |
| D_1_(h) | 0.101 |  |  |
| ALAG_1_(h) | 0.164 |  |  |
| CL/F or CL(L*h^-1^) | 7.39 | 6.1 | 4.73 |
| V(L) |  | 79.7 | 43.8 |
| Vc/F (L) | 10.9 |  |  |
| Vp/F (L) | 50.9 |  |  |
| Dose specific factor of F_1_ | 1(10 mg); 0.867 (15 mg); 0.608 (20 mg) |  |  |
| Impact of postprandial status on F_1_ | 0.244 |  |  |
| Impact of postprandial status on D_1_ | 4.90 |  |  |
| Impact of postprandial status on Ka | 0.830 |  |  |
| Age effect on CL/F |  | -0.011^c^ |  |
| CrCL on CL/F or CL | 1.84^a^ |  | 0.159^e^ |
| HCT1 on CL |  |  | -0.0132^f^ |
| SCrE on CL/F |  | -0.194^c^ |  |
| LBM effect on V/F |  | 0.01180^d^ |  |
| Impact of BMI on Vc/F | 1.36^b^ |  |  |
| Age effect on V/F (year^-1^) |  | -0.00133^d^ |  |
| proportional residual error | 21.0% | 47.9% | 13.1% |
| additive residual error | 1.87 |  |  |

Definitions of typical subject:

Our study：age=47.7 y; CrCL=97.2 ml/min; BMI=23.8 kg/m^2^；

ROCKET-AF model：age=65 years; SCR=1.05 mg dl^-1^; LBM=57 kg;

J-ROCKET-AF model: age=72 y; CrCL=65 ml/min; HCT1=42.2%;

a: Calculated as CL=exp (2.00409+ln (CRCL/95) * 0.61003)

b: Calculated as V=exp (2.392+ln (BMI/22.85) * 1.364)

c: Calculated as CL/F=6.10 × (1-0.011 × (Age-65) - 0.194 × (SCrE-1.09) )

d: Calculated as V/F=79.7 × (1-0.00133 × (Age-65) )+0.0118 × (LBM-57.5)

e: Calculated as CL=4.73 × ( CrCL / 67.11)^0.159^

f: Calculated as CL=4.73 × [1-0.0132 × (HCT1-42.14)]

**Supplementary Table S4 Comparison of model-based PT parameters of rivaroxaban across different models**

| Parameter | Our model | ROCKET AF model(1) | J-ROCKET AF model(2) |
| --- | --- | --- | --- |
| Baseline (s) | 11.4 | 11.4 | 11.4 |
| Slope(s/(ng/ml)) | 0.00180 | 0.043 | 0.0467 |
| Slope, describing decline of exponent on C_p_ (n/FACT) |  | 0.0000551 | 0.000155 |
| P_1_ | 1.37 |  |  |
| Covariates | BW, TCHO on BASE | CrCL on BASE, CrCL on decline of exponent | Age, LBM, ALBU, HB1 on BASE; TBIL on FACT |
| Proportional error model | - | 12.9% | 7.1% |
| Additive residual error (s) | 0.363 | - | - |

Structural model:

Our model: PT= BASE+SLOPE*C_P_^p1^

ROCKET AF model: PT= BASE+SLOPE*C_p_^(1-n×Cp)^

J-ROCKET AF model: PT= BASE+SLOPE*C_p_^(1-FACT×Cp)^


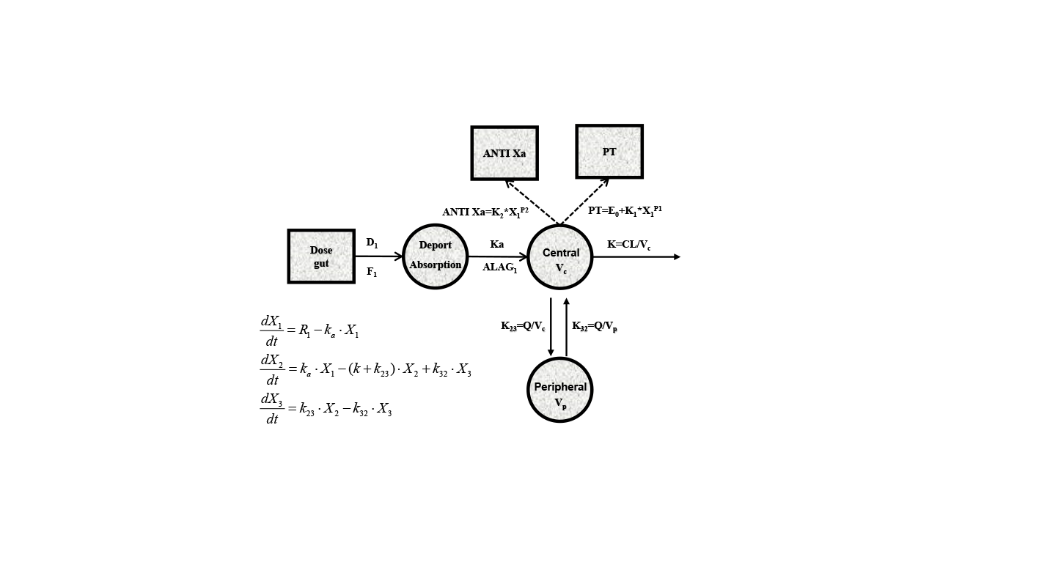


**Supplementary Figure S1 Final structure of the integrated PK/PD model.**

**
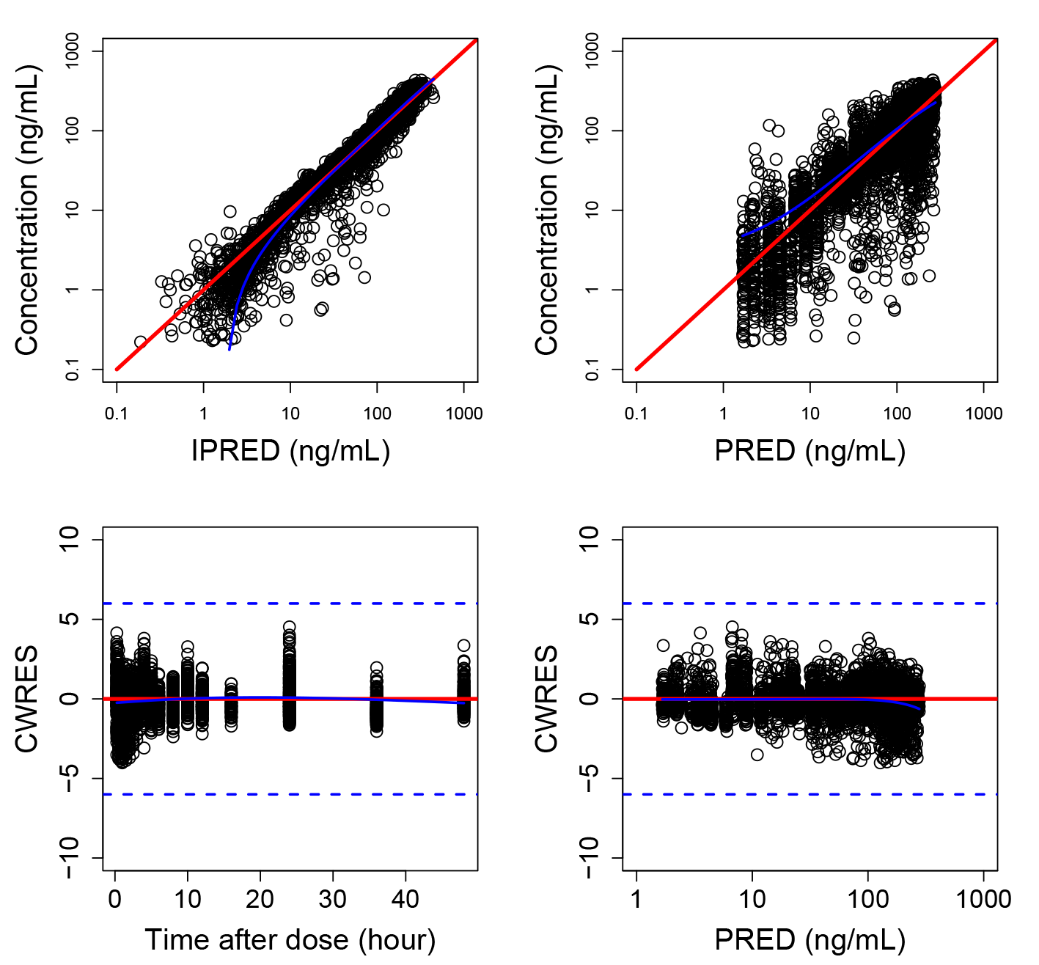
**

**Supplementary Figure S2 GOF plot for popPK model of healthy volunteers.**

**
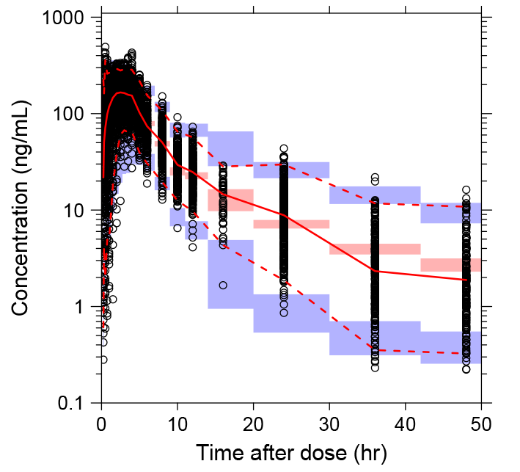
**

**Supplementary Figure S3 pc-VPC plot for popPK model of healthy volunteers.**

**
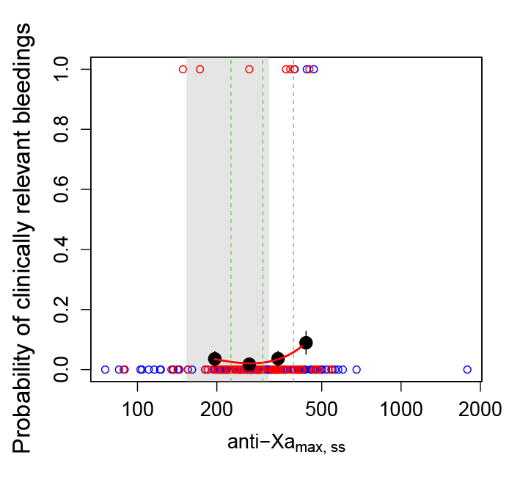

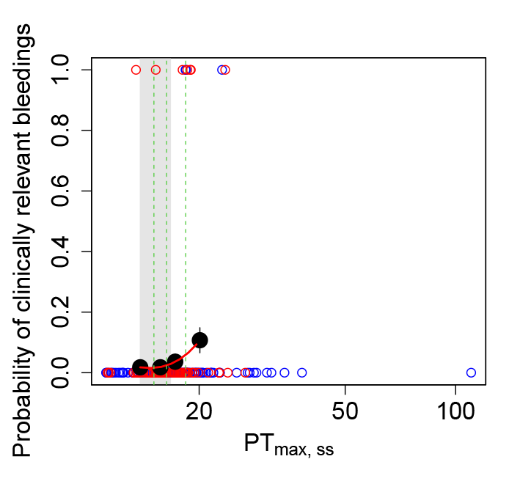
**

**Supplementary Figure S4 probability plot for clinically relevant bleeding and anti-Xa/PT.** The shaded area represents the expected anti-Xa/PT ranges converted from the estimated parameters at a steady-state (3).

**

**

**Supplementary Figure S5 Comparison plasma concentraion-time profile of different dosage.**

1. Girgis IG, Patel MR, Peters GR, Moore KT, Mahaffey KW, Nessel CC, et al. Population pharmacokinetics and pharmacodynamics of rivaroxaban in patients with non-valvular atrial fibrillation: results from ROCKET AF. *Journal of clinical pharmacology* (2014) 54(8):917-27. Epub 2014/03/29. doi: 10.1002/jcph.288. PubMed PMID: 24668660.

2. Kaneko M, Tanigawa T, Hashizume K, Kajikawa M, Tajiri M, Mueck W. Confirmation of model-based dose selection for a Japanese phase III study of rivaroxaban in non-valvular atrial fibrillation patients. *Drug Metab Pharmacokinet* (2013) 28(4):321-31. Epub 2013/01/23. doi: 10.2133/dmpk.dmpk-12-rg-109. PubMed PMID: 23337693.

3. Steffel J, Verhamme P, Potpara TS, Albaladejo P, Antz M, Desteghe L, et al. The 2018 European Heart Rhythm Association Practical Guide on the use of non-vitamin K antagonist oral anticoagulants in patients with atrial fibrillation: executive summary. *Europace* (2018) 20(8):1231-42. Epub 2018/03/22. doi: 10.1093/europace/euy054. PubMed PMID: 29562331.
